# Bone support correlation of X-Ray and CT for a new PE-glenoid

**DOI:** 10.1007/s00402-024-05556-3

**Published:** 2024-09-24

**Authors:** Matthias Bülhoff, Nikolai Sonntag, Raphael Trefzer, Bernhard Hirt, Sebastian Jäger, Mareike Schonhoff, Tobias Renkawitz, Philip Kasten

**Affiliations:** 1 Department of Orthopaedics, University Hospital Heidelberg, Schlierbacher Landstrasse 200a, Heidelberg, 69118 Germany; 2 Department of Orthopaedics Section of Biomechanics and Implant Research, University Hospital Heidelberg, Schlierbacher Landstrasse 200a, Heidelberg, 69118 Germany; 3 Institute for Clinical Anatomy and Cell Analysis, University of Tübingen, Elfriede-Aulhorn-Straße 8, Tübingen, 72076 Germany; 4 Orthopaedic Surgery Center (OCC), Wilhelmstr. 134, Tübingen, 72074 Germany

**Keywords:** Cementless glenoid, Anatomical shoulder arthroplasty, Cementless glenoid implant, Radiolucent lines, CT control cementless glenoid implant, Aseptic prosthetic loosening

## Abstract

**Introduction:**

The radiographic evaluation of novel cementless anatomic polyethylene (PE) glenoid components featuring a titanium-coated back is still unclear. This study explores potential radiolucent lines (RLL) between the radiopaque titanium layer and sclerotic convex reamed bone in an intermodal comparison analysis with computed tomography (CT) scans.

**Materials and methods:**

Eight RM pressfit vitamys glenoids (Mathys^®^) were implanted into cadaveric scapulae. In the CT scans, glenoids were quantified by evaluating ideal complete bony support (NO GAP) and gap between bone and titanium coating (GAP). X-rays were in perfect 0-degree projection and tilted in ± 10° and ± 20° mediolateral (ml) and craniocaudal (cc) directions. Radiographs evaluated were graded as NO RLL, RLL (gap > 1 mm) or DL (double line, gap < 1 mm) in an intermodal comparison of CT and X-ray findings.

**Results:**

The inter-rater (Cohen’s = 0.643) and intra-rater reliability (Cohen’s = 0.714) were good. The overall evaluation showed a significant agreement between (NO) RLL on X-ray and (NO) GAP on CT (*p* < 0.001). The − 10-degree ml projection showed good agreement between CT and X-ray (Cohen’s = 0.628). Adequate agreement was shown at 0 degrees (Cohen’s = 0.386), + 10 degrees ml (Cohen’s = 0.338), and + 20 degrees cc (Cohen’s = 0.327). Compared to the scenario DL = NO RLL, the true a.p. view showed better sensitivity when the DL is classified as RLL. Conversely, the true a.p. view demonstrated both better specificity and significant agreement between the X-ray and CT findings in scenario when DL = No RLL.

**Conclusion:**

Standard true a. p. projections are reliable in ruling out gaps when no RLL or DL is visible and the detection of RLL shows high intermodal agreement. Varying agreement across tilting angles emphasizes the importance of a comprehensive approach in evaluating bone support and CT is indispensable for a scientifically reliable assessment.

**Level of evidence:**

Level III Treatment Study.

## Introduction

Total shoulder arthroplasty (TSA) has become the gold standard for the treatment of end-stage degenerative diseases of the shoulder joint. The published results after TSA are promising. When treated for primary arthritis of the shoulder, TSA can lead to an improvement in the level of pain, mobility, and various scores in the short, medium and long term [1–3].

Results after TSA treatment with glenoid replacement are superior to shoulder hemiarthroplasty [4, 5]. However, loosening of the glenoid component remains a major problem in the long-term observation of TSA [6]. With the use of cemented glenoids, radiolucent lines (RLL) are a frequently observed phenomenon with a reported high prevalence between 0 and 96% [7]. Progressing RLL can possibly indicate implant loosening. The clinical relevance of stable RLL and its prognostic value regarding component failure is not yet known.

The results of cementless glenoid components described in the literature are still rare. However, higher complication rates were reported compared to cemented fixation techniques. These studies mainly concern cementless rigid metal-back glenoids [8]. The main advantage of modular metal-backed designs is the good ability to convert to a reversed prosthesis model without exchanging the entire glenoid component.

RLL around cementless rigid metal-back glenoid implants have been described with a prevalence of up to 70% when viewing conventional radiographs [7–11], which highlights the importance of using radiographic methods to assess implant loosening.

To reduce the incidence of RLL and radiographic loosening of the glenoid component, a new cementless PE glenoid with an elastic modulus similar to cancellous bone was introduced with porous titanium particle coating on the backside to improve osseointegration. To the best of our knowledge, there are still no data to radiographically evaluate the ideal fit of this PE implant in the glenoidal bone bed. Hypothetically, the thin radiopaque titanium layer on the convex PE component facing the sclerotic convex reamed bone could, in combination, simulate RLL.

As computed tomography (CT) provides higher sensitivity in the detection of implant loosening, it may need to be involved in the evaluation of radiological loosening signs that may have been obscured by projection radiographic examination [7, 9, 11].

These problems led to our investigation. This study investigates the extent to which the bony support of the titanium-coated PE glenoid in human specimens on CT scans corresponds to the presence of RLL or double lines (DL, gap < 1 mm) on radiographic images in different tilts.

## Materials and methods

### Specimen

Specimens were obtained from a university facility after informed consent. Exclusion criteria were malign disease, bone diseases or cysts, and metabolic diseases affecting the bone. All glenoids had a type A configuration, according to Walch [1]. The study was approved by the ethics committee of the local university (no. S-476/2022).

**Fig. 1:**
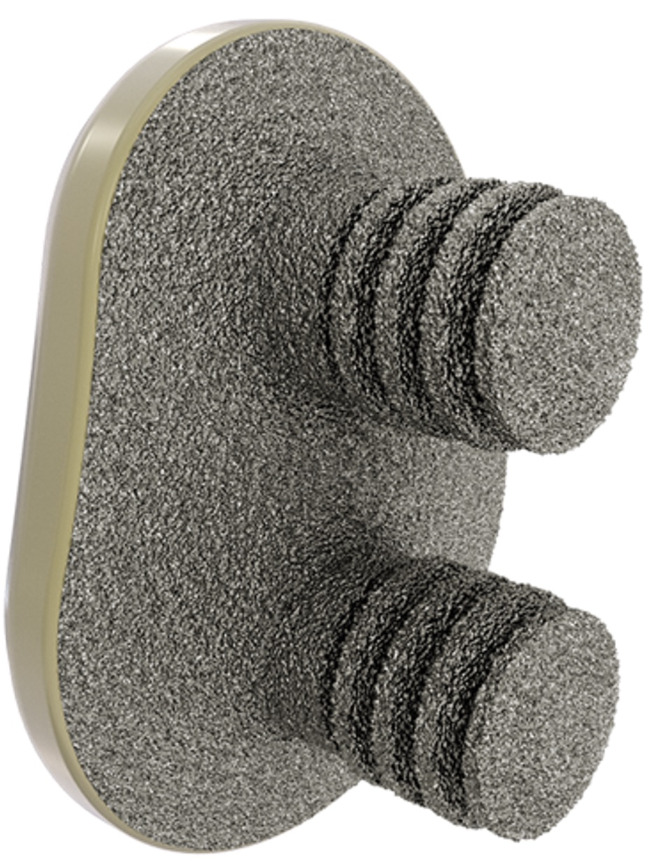
The glenoid component (Mathys^®^)

Eight RM pressfit vitamys glenoids (Mathys^®^) [6] (Fig. 1) were implanted into cadaveric scapulae after orthograde embedding in epoxy resin for fixation. All parts that we treated surgically were excluded and not embedded in epoxy resin. Fixation in epoxy resin enabled us to ensure a standardized surgical treatment and radiological analysis procedure. Implantation surgery was performed by an experienced shoulder surgeon (PK) according to the manufacturer’s manual.

The cementless titanium-coated glenoid implant component with an elastic modulus similar to cancellous bone is available in 4 sizes and has a convex back.

### Size templating

The size of the glenoid implants was determined using true a.p. radiographs, and templating was performed using the Brainlab Traumacad software (München, Germany). In all cases, the planning size matched the selected implant size in the lab.

### Surgical technique

**Fig. 2:**
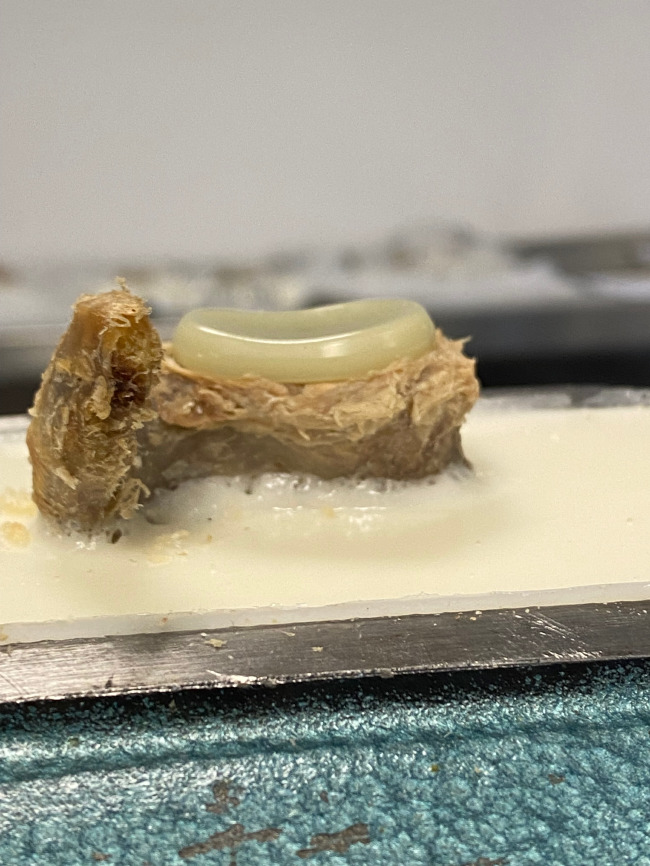
The glenoid implant after impaction into a human cadaveric specimen

According to the surgical manual, the central K-wire was inserted into the glenoid using the appropriate template. This was followed by reaming with the appropriate planned size up to the beginning of the subchondral bone. The drilling template for the Pegs was then applied over the K-wire and the holes for the Pegs were drilled. The glenoid implant was then inserted according to the measured size (Fig. 2).

### Image evaluation

In the postoperative CT scan, the glenoid was evaluated in the coronal cutting plane regarding ideal complete support (NO GAP), a gap between bone and titanium coating (GAP ≥ 1 mm) and double lines (DL; GAP < 1 mm).

For the X-ray evaluation, radiographs were taken in perfect true antero-posterior (true a.p.) projection. An ideal alignment with the X-ray beam has taken place using a 3D-printed fitting. Additionally, the glenoid was tilted in ± 10° and ± 20° mediolateral (ml) and craniocaudal (cc) directions, leading to a total of 72 X-ray images. The X-ray images were assessed twice by two observers (MB and PK; experienced shoulder surgeons) at an interval of 6 weeks using the Miele-LXIV program and evaluated regarding NO RLL, RLL (GAP ≥ 1 mm) or DL (Double Line, GAP < 1 mm).

**Fig. 3:**
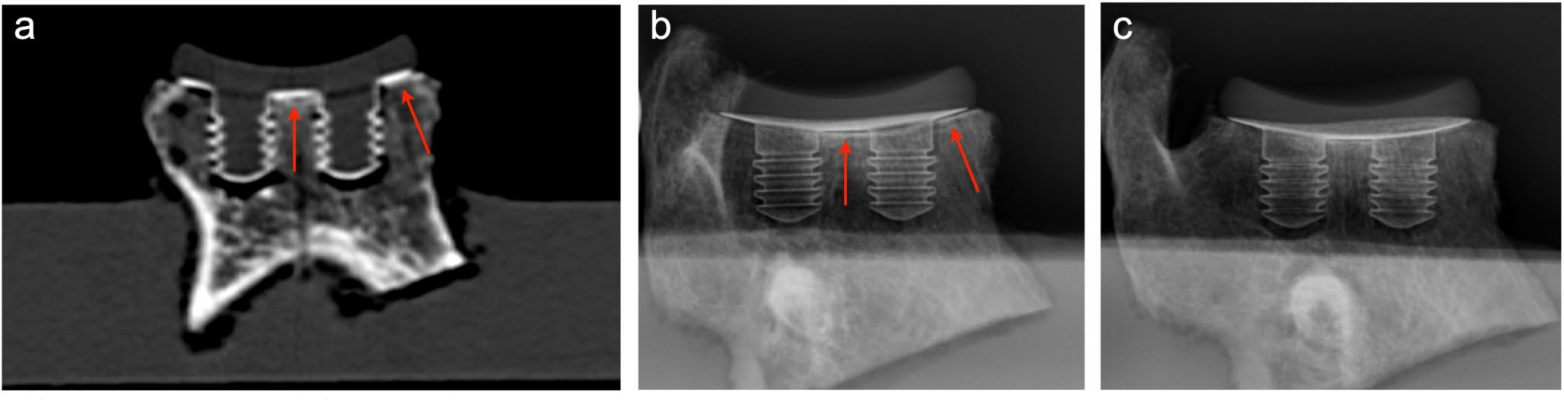
Different quantities in bone support after implantation surgery in the CT scan and on conventional X-rays (**a**) CT scan of the coronal cutting plane shows no gap between the coated PE glenoid and bone. (**b**) Conventional true a. p. radiograph displaying a DL in zones 3 and 5. (**c**) -10-degree craniocaudal (cc) tilted a. p. radiograph showing no signs of RLL or DL

Finally, the CT scan and X-ray findings were compared. It was examined whether classifying the DL as RLL or as NO RLL led to a higher intermodal agreement between CT and X-ray findings (Fig. 3).

Agreement was categorized as follows: Cohen’s κ > 0.2 sufficient agreement; > 0.4 moderate agreement; > 0.6 good agreement.

### Statistical analysis

The effect size was planned using G*Power version 3.1.9.6 based on the data from a previous publication [2]. The effect size d was calculated based on the radiologically measured group parameters. The measured sample size was 7. For an effect size of d = 1.5 and a statistical power of 0.9, eight test specimens were investigated. The data were systematically aggregated into a coherent table within Microsoft Excel^®^ (Version 16.55, 2021) for statistical analysis. Subsequently, the statistical evaluation was conducted utilizing the SPSS^®^ software for Windows, Version 22.0 (SPSS Inc., Chicago, Illinois, USA). The inter- and intra-rater reliability was determined using Cohen’s Kappa index based on the results of two examiners over a period of 4 to 6 weeks.

Data were tabulated per group and statistical analysis was performed using SPSS Software (IBM Corp., Armonk, NY, USA).

Agreement was defined as follows: Cohen’s κ > 0.2: sufficient agreement; > 0.4 moderate agreement; > 0.6 good agreement.

Significance level was set to *p* < 0.05.

## Results

The inter-rater (Cohen’s κ = 0.643) and intra-rater reliability (Cohen’s κ = 0.714) of radiograph evaluation regarding NO RLL, RLL or DL between the two highly experienced observers were good.

Correlation between CT scan and X-ray images:

The overall evaluation showed a significant agreement between (NO) RLL on X-ray and (NO) GAP on CT (*p* < 0.001).

The 0-degree true a.p. image showed significantly higher sensitivity when the DL was classified as RLL (81.3%). Conversely, if a DL was defined as NO RLL, better specificity could be achieved in the true a.p. image (97.9%), but at a considerable expense in sensitivity (50.0%) (Table 1). When DL was classified as NO RLL, there was a moderate significant agreement for the true a.p. radiographs, but only sufficient significant agreement when DL was classified as NO RLL.

For the craniocaudal (cc) tilted projections, moderate significant agreement was observed for − 20° cc (sensitivity: 81.3%; specificity: 88.2%), -10° cc (sensitivity: 81.3%; specificity: 90.3%) and + 10° cc (sensitivity: 75.0%; specificity: 85.4%) projections and sufficient significant agreement for + 20° cc (sensitivity: 62.5%; specificity: 84.7%), when DL were classified as RLL. When DL were classified as NO RLL, good significant agreement was reached for − 20° cc (sensitivity: 56.3%; specificity: 98.6%) and − 10° cc (sensitivity: 56.3%; specificity: 98.6%) projections, whereas + 10° cc (sensitivity: 31.3%; specificity: 95.8%) and + 20° cc (sensitivity: 25%; specificity: 95.8%) projections displayed sufficient significant agreement (Table 1).

In the medio-laterally (ml) tilted projections, RLL was not detectable in -20° ml projections and could not be analyzed. When DL was classified as RLL, -10° ml projections showed good significant agreement (sensitivity: 68.8%; specificity: 95.8%), + 10° ml showed sufficient significant agreement (sensitivity: 62.5%; specificity: 85.4%), and + 20° ml projections showed moderate significant agreement (sensitivity: 37.5%; specificity: 97.9%). When DL was classified as NO RLL, -10° ml projections showed sufficient significant agreement (sensitivity: 12.5%; specificity: 100%), + 10° ml projections showed moderate significant agreement (sensitivity: 31.3%; specificity: 99.3%) and + 20° ml projections showed sufficient significant agreement (sensitivity: 25%; specificity: 100%) (Table 1).

**Table 1:** Shows the sensitivity and specificity for different tilts of the radiographic projections

X-ray projection, which is compared to CT	DL = RLL				DL = NO RLL			
	Sensitivity (GAP + RLL)	Specifity (NO GAP + NO RLL)	Cohen’s Kappa		Sensitivity (GAP + RLL)	Specifity (NO GAP + NO RLL)	Cohen’s Kappa	
			Index	*p*-value			Index	*p*-value
0°	81,3%	81,9%	0,368 (sufficient)	< 0.001	50,0%	97,9%	0,556 (moderate)	< 0.001
-20° cc	81,3%	88,2%	0,500 (moderate)	< 0.001	56,3%	98, 6%	0,637 (good)	< 0.001
-10° cc	81,3%	90,3%	0,548 (moderate)	< 0.001	56,3%	98,6%	0,637 (good)	< 0.001
+10° cc	75,0%	85,4%	0,410 (moderate)	< 0.001	31,3%	95,8%	0,315 (sufficient)	< 0.001
+20° cc	62,5%	84,7%	0,327 (sufficient)	< 0.001	25%	95,8%	0,250 (sufficient)	0.001
-20° ml	-	100%	RLL not visible, not applicable for analysis		-	100%	RLL not visible, not applicable for analysis	
-10° ml	68,8%	95,8%	0,628 (good)	< 0.001	12,5%	100%	0,205 (sufficient)	< 0.001
+10° ml	62,5%	85,4%	0,338 (sufficient)	< 0.001	31,3%	99,3%	0,423 (moderate)	< 0.001
+20° ml	37,5%	97,9%	0,440	< 0.001	25%	100%	0,375 (sufficient)	< 0.001

## Discussion

This study investigates the correlation between CT scan and conventional radiographic findings for the assessment of RLL and DL in a new cementless, titanium-coated glenoid component in shoulder arthroplasty. To the best of our knowledge, the current literature does not yet contain data specific to this issue.

The current findings support the idea that double lines and RLL should be judged as RLL. If surgeons see an RLL on the postoperative X-ray, they get the feedback that the glenoid has not perfectly been implanted with respect to a 100% bone contact between titanium and bone.

If no RLL or DL is visible on the X-ray, it can be assumed that the PE is in direct contact with the bone. The evidence of RLL or DL also showed a high degree of agreement between X-ray and CT findings.

However, the 0-degree projection, as the supposed “gold standard” in daily clinical practice, only shows sufficient to moderate agreement between X-ray and CT scan assessments.

Alternatively, repeated X-ray fluoroscopy at -10 degrees and − 20 degrees cc tilt could assist the surgeon to determine whether the appearance of RLL corresponds to the presence of a gap, as these projections showed better agreement. A gap may be likely if an RLL occurs in these projections, even if it is strictly a DL. However, in daily practice, the projection in the X-ray setting can hardly be adjusted with an accuracy of 10 degrees of tilt. Across all projections, a DL, i.e. an RLL < 1 mm, should be defined as RLL in order not to underestimate gaps between the implant and bone. Overall, the sensitivities of radiographs to detect gaps were low. A CT scan, as the gold standard to detect RLL, should be added whenever RLL is observed on an X-ray or in cases of doubt [12]. A recent study by R. Emery examined micromotions and the appearance of RLL in CT scans of patients who were treated with a total shoulder arthroplasty with a cemented keel glenoid [13]. In all 7 patients, RLL could be detected in the CT scan after 24 months. This supports the high importance of CT scan diagnostics in detecting RLL over time and is in accordance with our findings.

At least the RLL should be followed-up and not underestimated. However, we do not know the impact of our findings on clinical outcome parameters. No studies known to us evaluate the results of patients treated with a cementless, titanium-coated glenoid component.

The implant design of a cementless fixation technique with a titanium-coated polyethylene implant is not new and is well-described for hip arthroplasty surgery [3, 4].

The advantages of such a glenoid component design with a thin titanium coating are the cementless fixation technique, the bone-preserving aspect, and avoiding an over-stuffing of the joint [7]. An issue in conventional rigid metal-back glenoids is over-stuffing due to the thickness of the metal-back and the PE insert. This might explain the high rates of rotator cuff deficiencies as a consequence of over-tensioning the tendons.

In anatomic shoulder arthroplasty, loosening of the glenoid component remains one of the major problems compromising long-term implant survival.

RLL around the glenoid component can be a possible sign of implant loosening. However, the phenomenon of RLL is still not fully understood but well-described. The occurrence of radiolucent lines (RLL) in cemented PE glenoids has been reported with up to 96% [8]. Cement pressurization led to a lower rate of RLL in a prospective randomized study in the short term [9]. In a monocentric study of over 900 patients, survival rates of 92% after 15 years were described [14]. Another study showed a survival rate of almost 98% after 10 years [15]. These are good results. The new glenoid designs will have to compete with these findings in the future.

On the other hand, the occurrence of RLL is not inevitably associated with loosening of the glenoid component or clinical symptoms [10, 11].

In cementless glenoid replacement, the results of rigid metal-backed concepts are mainly described. High mid-term failure rates are reported [16, 17]. A reason for this could be the aforementioned over-stuffing, instability or backside wear of the modular glenoids. The New Zealand, Australian, and British TSA registries also support this finding in the long run.

One model shows promising results: in a study by A. Castagna’s working group, none of the glenoid components were loosened after an average of 6 years in 35 patients with a cement-free glenoid [18]. Questions have been raised regarding a selection bias in this study: the patients were selected over a long period, and the glenoid morphology showed minor severity according to Walch. Long- term results from this study from 2010 have not been published yet.

In another investigation, Schoch et al. presented early clinical and radiological results of patients treated with a hybrid ingrowth cage polyethylene glenoid component. The Pegs of the component have a cage design to allow bony ingrowth. At a follow-up of 33 months, 51 shoulders were examined. Almost one-fourth showed RLL in the radiological follow-up. The clinical outcome parameters were good [19]. The main difference is the implant design used in the study of Schoch compared to the implant used in this study.

However, the literature about non-metal-backed cementless glenoid replacement is sparse, and long-term results are not yet available.

Mechanisms that can lead to loosening of cemented glenoidal components are described in multicenter studies. A medial migration of the component (= subsidence) as well as a cranial and dorsal tilt (= tilt) describe a common radiological correlate of loosening. Risk factors are aggressive reaming, inaccurate implant positioning, and dorsal subluxation of the humeral head [15, 20, 21]. Whether these aspects will also have an effect on cementless glenoid components, remains unclear for now.

Comparable studies on cement-free glenoid implants, as examined here, are not available yet in the literature. However, excessive and aggressive reaming into the subchondral bone should be avoided, especially with cement-free implant designs. Vice versa, reaming that is too conservative can leave gaps between the implant and the bone. Nevertheless, perfect osseointegration of the implant can result over time, even in imperfectly set implants. Osseointegration will most likely occur around the Pegs, in particular, due to much less micromotion compared to the area right behind the PE glenoid. It is assumed that perfectly set components will result in better clinical outcomes and be associated with a lower risk of component loosening over time. Here, our findings give valuable information at a time zero right after implantation.

Further comparative studies are necessary to explore whether the novel cement-free implant designs, as examined in this study, can survive in the long term and compete with the established glenoid component models.

Limitations of this study are the low number of cases and the fact that it is a time-zero cadaver study under ideal conditions. Accordingly, we cannot give information about the long-term clinical relevance of our findings. Further investigations need to address that aspect.

## Conclusion

Standard true a. p. projections are reliable for ruling out gaps when no RLL or DL are visible. Specificity is up to 97.9% and the detection of RLL shows high intermodal agreement. However, overall sensitivities of radiographs to detect gaps were low. A CT scan might help to quantify RLL and DL and should be performed in daily clinical follow-up practice whenever RLL is observed on an X-ray.

Mathys^®^ (Bettlach, Switzerland) supported this study financially by providing the instrumentation and glenoid implants.
